# Molecular tagging of seed size using MITE markers in an induced large seed mutant with higher cotyledon cell size in groundnut

**DOI:** 10.1007/s13205-023-03909-0

**Published:** 2024-01-29

**Authors:** Poonam Gajanan Bhad, Suvendu Mondal, Anand M. Badigannavar

**Affiliations:** 1 Nuclear Agriculture and Biotechnology Division, Bhabha Atomic Research Centre, Mumbai, 400085 India; 2 Homi Bhabha National Institute, Training School Complex, Anushaktinagar, Mumbai, 400094 India

**Keywords:** *Arachis hypogaea*, Cell size, Linkage map, Large seed mutant, Quantitative trait loci

## Abstract

A large seed mutant, TG 89 having a 76.7% increment in hundred kernel weight in comparison to its parent TG 26, was isolated from an electron beam-induced mutagenized population. Studies based on environmental scanning electron microscopy of both parent and mutant revealed that the mutant seed cotyledon had significantly bigger cell size than parent. A mapping population with 122 F_2_ plants derived from the mutant and a distant normal seed genotype (ICGV 15007) was utilized to map the QTL associated with higher HKW. Bulk segregant analysis revealed putative association of three markers with this mutant large seed trait. Further, genotyping of F_2_ individuals with polymorphic markers detected 14 linkage groups with a map distance of 1053 cM. QTL analysis revealed a significant additive major QTL for the mutant large seed trait on linkage group A05 explaining 12.7% phenotypic variation for the seed size. This QTL was located between flanking markers AhTE333 and AhTE810 having a map interval of 4.7 cM which corresponds to 90.65 to 107.24 Mbp in A05 chromosome, respectively. Within this genomic fragment, an ortholog of the *BIG SEEDS 1* gene was found at 102,476,137 bp. Real-time PCR revealed down-regulation of this *BIG SEEDS 1* gene in the mutant indicating a loss of function mutation giving rise to a large seed phenotype. This QTL was validated in 11 advanced breeding lines having large seed size from this mutant but with varied genetic backgrounds. This validation showcased a highly promising selection accuracy of 90.9% for the marker-assisted selection.

**Supplementary Information:**

The online version contains supplementary material available at 10.1007/s13205-023-03909-0.

## Introduction

Groundnut (*Arachis hypogaea* L.), although classified as a legume, stands out as an oilseed crop which is rich in essential nutrients like proteins, healthy fats, and other vital nutrients like vitamins, antioxidants, polyphenols, phytosterols, fibre, vital vitamins and minerals, it is a crucial component in addressing global hunger and ensuring proper nutrition for consumers (Settaluri et al. 2012; Zhou et al. 2021). Owing to its nutrient-dense nutty texture and affordability, it is also referred to as poor man’s nut, fetching premium price for large kernels. This led to an increase in direct consumption of groundnut to 50% of its total usage and led to a downward shift in a portion of seeds processed for oil extraction from 42% to under 23% (Sharma 2017). Groundnut's dual role as an oilseed and versatile food ingredient addresses nutritional, economic and environmental aspects, making it vital in modern agriculture and food production. A rise of 23.7% was logged in the total area harvested in 2021 than that in 2020 (FAOSTAT 2021). On a global scale, groundnut cultivation spans approximately 37.7 million hectares, yielding a production of 72.3 million tonnes and an average productivity of 1917.7 kg per hectare (FAOSTAT 2021). India stands out as a major contributor, with 15.8% of the total harvested area dedicated to groundnut cultivation. Notably, countries like India, China, the USA, and Nigeria collectively account for over 75% of the world's groundnut production. In India alone, groundnut is cultivated across 5.97 million hectares, resulting in a total production of 10.2 million tonnes and a yield of 1718.2 kg per hectare (FAOSTAT 2021).

Groundnut is internationally valued for its oil-rich seeds and protein content, enjoyed in a variety of culinary and confectionery forms, with its diverse kernel sizes being particularly desirable for confectionary and direct consumption. Large seed size in groundnuts not only impacts both yield and market value but also provides several advantages in agricultural production and marketability. In terms of agricultural yield, larger seeds exhibit increased germination rates and early seedling vigour, leading to better crop establishment (Iledun and Patience 2020). They also result in higher pod setting, contributing to increased pod yield (Patil 1975; Kale et al. 2000; Gomes and Lopes 2005; Venuprasad et al. 2011). Many studies have indicated a positive correlation between seed size and pod yield (Sah et al. 2000; Venkataramana et al. 2001; Lakshmidevamma et al. 2004). Moreover, large seeds show resilience to stress factors like drought and diseases, leading to more stable crop production (Chu et al. 2020). Thus, seed size being an important characteristic for the groundnut industry remained under strong selection pressure towards breeding for higher seed size and yield.

Seed size is frequently measured as hundred kernel weight (HKW) and is a crucial selection parameter in developing groundnut varieties of confectionary value (Kale et al. 2000; Venuprasad et al. 2011). Overall seed size is a quantitative trait that is controlled by polygenes (Garet 1976; Layrisse et al. 1980; Swe and Branch 1986; Anderson et al. 1993), trigenes (Pattanashetti et al. 2008) and both maternal and nuclear genes (Hariprasanna et al. 2008; Venuprasad et al. 2011). Many studies have reported the occurrence of large seed mutants in groundnuts in the past. A large seed mutant was induced in groundnut using X-rays (Patil 1966), gamma rays (Reddy et al. 1977; Branch 2002; Badigannavar and Murty 2007; Badigannavar et al. 2020) and also using sodium azide (Hussain et al. 1991; Wang et al. 2002).

Genetic linkage mapping has elucidated number of quantitative trait loci (QTL) for HKW, seed length and width using bi-parental mapping populations derived from hybridization among existing germplasm (Shirasawa et al. 2012; Fonceka et al. 2012; Huang et al. 2015; Chen et al. 2016; Hake et al. 2017a; Alyr et al. 2020; Chavarro et al. 2020; Wang et al. 2022a). Luo et al. (2017, 2018) reported major QTLs for the hundred-pod weight (HPW) localized on chromosomes A05 and A07 in groundnut. Chu et al. (2020) identified a major QTL for seed size in the A05 chromosome (93–102 Mbp) explaining 66% phenotypic variation. Alyr et al. (2020) reported one significantly associated QTL for pod size on A07. Chavarro et al. (2020) reported QTL for seed and pod weight on A07, B02 and B06 chromosomes. Zhang et al. (2019) identified QTLs for hundred seed weight on A02 and B06 in groundnut. Gangurde et al. (2020) discovered several major QTLs related to pod and seed weight on chromosomes A05, A06, B05, and B06 in two nested association mapping (NAM) populations of groundnut. Genome wide association mapping has also uncovered significant marker trait associations for HKW and seed length in groundnut (Pandey et al. 2014; Zhao et al. 2017; Zou et al. 2020). Using differential gene expression studies, Wan et al. (2017) identified two candidate genes encoding cinnamyl alcohol dehydrogenase (CAD) and 1-aminocyclopropane-1-carboxylate synthase (ACS) which regulated the pod size in *pw* mutant in groundnut. Wang et al. (2022b) have reported the synergistic role of plant hormones indole-3-acetic acid, gibberellin, and brassinosteroid in governing pod size mainly by affecting cell expansion in groundnut. The heterologous overexpression of an auxin-regulated gene from rice, *OsARGOS*, had increased both cell size as well as cell number in *Arabidopsis* resulting in large leaves as well as enlarged siliques (Wang et al. 2009). A loss of function mutation in the *ARF18* encoding an auxin-response factor induced longer siliques by speeding up cell expansion in *Brassica napus* L. (Liu et al. 2015). Overexpression of the *TaCYP78A3* gene was positively associated with the seed size in wheat (Ma et al. 2015). Loss of function mutation in the *BIGSEEDS 1* (*BS1*) gene has been shown to control seed size and pod size in *Medicago trunculata* and soybean (Ge et al. 2016). Map-based cloning of large seed mutant, *da 1-1* revealed that a DA1 protein (coding for ubiquitin receptor protein) regulates seed size by increasing the duration of cell proliferation in *Arabidopsis* (Li et al. 2008). Wang et al. (2016) identified a dysfunctional STERILE APETALA-like protein (SAP) in a mutagenized population of *da 1-1* mutant that suppressed the phenotype of *da 1-1*. This SAP protein positively controls flower, seed, and leaf size in *Arabidopsis* by increasing cell proliferation but does so independently of the DA1 protein (Wang et al. 2016). This SAP protein influences the stability of transcriptional regulators like PEAPODs (PPDs) (Li et al. 2018). Reports on seed size QTL of mutant origin in groundnut is almost absent in the literature. Mutants for such polygenic traits of agronomic importance could be a very good source for the identification of large effect QTLs and thereby their utilization in marker-assisted breeding programs.

The present study makes use of a large seed mutant that was isolated from an electron beam-induced mutagenized population of TG 26 (Mondal et al. 2017). Preliminary inheritance of this large seed mutant revealed that it was controlled by a recessive gene (unpublished data). The oligogenic nature of this large seed mutant trait has prompted us to tag this mutant trait with molecular markers. Due to the very limited polymorphism between TG 26 and its mutant (TG 89), its F_2_ population will not be suitable to use for tagging the mutant trait through conventional mapping. Hence, the mutant TG 89 was hybridized with a distant breeding line ICGV 15007 and its F_2_ population was used for molecular tagging of large seed trait in this mutant. In the present study, we report the anatomical behaviour of cotyledonary cells of mutant, identification of a major QTL for HKW through bulk segregant analysis, QTL mapping of seed size trait, and gene expression of the putative candidate gene that contributes to genetic variation for mutant large seed trait in cultivated groundnut.

## Materials and methods

### Plant material and phenotyping

A large seed mutant TG 89 was isolated from an electron beam-induced population of TG 26 (Mondal et al. 2017). The HKW for TG 26 and TG 89 was 45.5 ± 3.1 g and 80.4 ± 3.1 g, respectively. TG 26 is a mutant-derivative variety from the hybridization between BARCG 1 (Mutant of JL 24) and TG 23 (Kale et al. 1997). The large seed mutant, TG 89 has 76.7% larger seed size than its parent, TG 26. TG 89 (large seed) was crossed with ICGV 15007 (normal seed) to develop an F_2_ mapping population consisting of 122 lines. This distant parent, ICGV 15007 was obtained from the International Crops Research Institute (ICRISAT), Patancheru, India and has high oleic acid (80%) in its seed oil. ICGV 15007 is a breeding line developed from the cross of ‘ICGV 06420 X Sun Oleic 95R’. The F_2_ population was phenotyped for HKWs in the rainy season, 2019 at the Experimental and Gamma Field Facility, Bhabha Atomic Research Centre, Trombay, Mumbai, Maharashtra, India.

### Environmental scanning electron microscopy

Environmental scanning electron microscopy (ESEM) was employed for the analysis of cell size at various time points of pod development in the mutant and its parent. To understand the histological development of large seed, the developing pods were harvested at 30, 40, 50 and 60 days after fertilization (DAF) and stored in formalin solution at -20° C until further analysis. The samples were air-dried in a desiccator and a thin cross-section of three to five mm of the samples was fixed to the double adhesive tape of the aluminium stubs and the surfaces were directly observed under the environmental scanning electron microscope (FEI, Quanta 200, Netherlands) under low pressure of 65 Pa and beam energy of 15 kV. The cell size was measured from SEM images by ImageJ software (Schneider et al. 2012).

### Genomic DNA isolation

Genomic DNA was extracted using a GeneJET Plant Genomic DNA Purification Mini kit (Thermo Fisher Scientific, Vilnius, Lithuania). For this, 100 mg of fresh leaflet was excised from a 30 days old plant and grounded to fine powder in liquid nitrogen. The ground powder was immediately transferred to the 2 ml microcentrifuge tube with 350 μL lysis buffer A and 10 μL of β-mercaptoethanol to prevent oxidation. Next, 50 μL of lysis buffer B and 20 μL RNase A were added, and the sample was incubated at 65°C for 30 min, in a water bath. After incubation, 130 μL of precipitation solution was added, and the mixture was incubated on ice for 15 min. It was then centrifuged to collect the supernatant which was later mixed with 400 μL plant gDNA binding solution and 400 μL 96% ethanol. This mixture was applied to a spin column and centrifuged in two batches as per manufacturer’s instructions. The spin column was washed twice with wash buffer I and wash buffer II and finally, the purified DNA was eluted with 50 μL elution buffer. The isolated DNA was quantified by UV–Vis spectrophotometer (Jasco, Japan) at 230, 260 and 280 nm. The A_260_/A_280_ ratio was also considered to check protein contamination and the A_260_/A_230_ ratio was used to indicate the presence of unwanted organic compounds. Principally, for a good quality DNA, the A_260_/A_280_ ratio should be in the range of 1.8–2.0 and A_260_/A_230_ ratio should be in the range of 2.0–2.2. Based on the OD at 260 nm reading of the sample on a spectrophotometer, the concentration of DNA was calculated using following formula: DNA concentration = 50 μg/mL × OD260 × dilution factor. The resulting purified DNA was ready for downstream applications or can be stored at −20 °C. Finally, as per to the concentration present, the DNA was diluted with sterile deionized water to get the final concentration of DNA of 10 µg/ml for use in genotyping experiment.

### Bulk segregant analysis (BSA) and testing of marker polymorphism

The modified bulked segregant analysis (BSA) was used to identify miniature inverted transposable element markers (MITE) and simple-sequence repeat (SSR) markers linked to HKW (Michelmore et al. 1991). In this modified BSA, large seed bulk and normal seed bulks were prepared by pooling 10 ng of genomic DNA from 10 F_2_ plants together each having the highest HKW value and the lowest HKW value, respectively. Both MITE and SSR primers that showed polymorphism in parents as well as in corresponding bulks, were used for genotyping 122 F_2_ plants for the construction of a genetic linkage map. Besides, 85 polymorphic markers were also used to amplify the entire F_2_ population towards making a coarse linkage map. For DNA amplification, a 10 µl reaction volume contained 10 ng template DNA, 10 mM Tris–HCl (pH 9.0), 1.5 mM MgCl_2_, 200 µM of each dNTPs, 0.2 µM of each forward and reverse primer and 0.5 U Go *Taq* DNA polymerase (Promega, WI, USA). DNA amplification with each of the primers was performed in a thermal cycler (peqSTAR Thermocycler, VWR, Germany) using the following conditions: 94 °C for 5 min, 10 cycles of 94 °C for 30 s, 55 °C (with decrement in temperature at −0.5 °C per cycle) for 30 s, extension at 72 °C for 45 s; followed by 25 cycles of denaturation at 94 °C for 30 s, annealing at 55 °C for 30 s, extension at 72 °C for 45 s and a final extension step at 72 °C for 10 min. The amplified PCR products from AhMITE markers were separated in 1.5% agarose gel using electrophoresis (Sigma, MO, USA) with 0.1% ethidium bromide for visualization in UV light. Gel images were captured using a gel documentation system (Kodak, NY, USA). The amplified products of SSR markers were loaded onto a capillary gel electrophoresis-based platform and the amplicon size was derived with reference to a suitable size marker (QIAxcel Advanced, Qiagen, Germany).

### Linkage analysis

Each new SSR and MITE marker reaction in the F_2_ mapping population was tested for a 1:2:1 or 3:1 segregation ratio using the *χ*^2^ test based on the codominant and dominant behaviour of markers, respectively. The linkage map was constructed using QTL IciMapping ver 4.1 (Meng et al. 2015). Markers having a LOD threshold more than 3 and a maximum map distance of 37.2 cM were grouped together. Marker order in a linkage group was deciphered using ‘nnTwopoint’ analysis. ‘nnTwoOpt’ algorithm is heuristic but not exact therefore, after ordering each marker sequence, rippling was done for fine-tuning with the ‘SAD’ (sum of adjacent distances) algorithm. The map genetic distance was calculated using the Kosambi map function and expressed in centiMorgan (cM) (Kosambi 1944).

### QTL analysis for HKW

To identify the main QTLs, phenotypic values of HKW data of each of the F_2_ individual plants were entered along with the marker genotypic values in Inclusive Composite Interval Mapping (ICIM) analysis (Li et al. 2007). Regression analysis was carried out using ICIM-ADD application in QTL IciMapping version 4.1 (Meng et al. 2015). Additive QTL was then located using a scanning window of 1.0 cM by following a stepwise regression method. The accurate “logarithm of the odds” (LOD) thresholds was calculated using one thousand permutations with a type I error probability set to 0.01. A QTL having LOD value higher than this threshold was designated as a significant additive QTL. A LOD is a statistical estimate of the relative probability that two loci are located near each other on a linkage group and are therefore likely to be inherited together. A higher LOD score indicates stronger evidence for linkage, making it more significant. (Lander and Kruglyak 1995). Graphic visualization of linkage groups and identified QTLs were designed in Mapchart version 2.1 (Voorrips 2002).

### Gene expression assay using a quantitative RT-PCR

Total RNAs of groundnut leaves were extracted from TG 26 and TG 89 using the TRIzol method and purified using Spectrum Plant Total RNA Kit (Sigma, St. Louis, USA). A total of 1 μg RNA were used to synthesize the first-strand cDNA using a Takara PrimeScript cDNA synthesis kit (Takara, Japan) according to the manufacturer's instructions using oligo-dT primers, RNase inhibitor and PrimeScript Reverse Transcriptase. Gene-specific primers consisting of left primer 5′ GGAGAAGCGAAAGGAAAGGG 3′ and right primer 5′ TGGTAGCGAACAGGAACCG 3′, for gene a*rahy.5M7JWE* (BS1 homolog in groundnut) were designed and the *G6PD* gene (Reddy et al. 2013) was used as the internal control. Quantitative RT-PCR was performed on the Rotor Gene Q machine (Qiagen, Germany). qRT-PCR was performed in a reaction volume of 10 μL containing 6 μL of 2 × SYBR Green KiCqStart SYBR Green qPCR ready mix (Sigma-Aldrich), 1 μL of each of forward and reverse primers (10 mM), and 4 μL cDNA template (diluted 1:10). All samples were run in duplicate. The real-time PCR conditions were as follows, pre-denaturing at 95 °C for 5 min, followed by 40 cycles of (denaturation at 94 °C for 15 s, annealing at 55 °C for 20 s and extension at 72 °C for 20 s) and fluorescence emissions were measured at the extension step of each of the repetitive cycles. The nonspecific products were identified using the melt curve analysis feature of rotor gene Q. The fold change expression levels of the target genes were calculated using the 2^−ΔΔCt^ method using internal control of a housekeeping gene, *G6PD* (Livak and Schmittgen 2001).

### Validation of linked markers

Eleven advanced breeding lines (at *F*_7_ generation) from the crosses involving mutant were selected in successive generations for their yield and higher HKW. These were grown during the Summer 2022 in two replications to measure HKW and per se performance. Genomic DNA was extracted from young leaflets and tested for the presence of amplified products of QTL flanking markers.

## Results and discussion

### Larger seed size in mutant is due to increased cell size in seed cotyledon

**Fig. 1:**
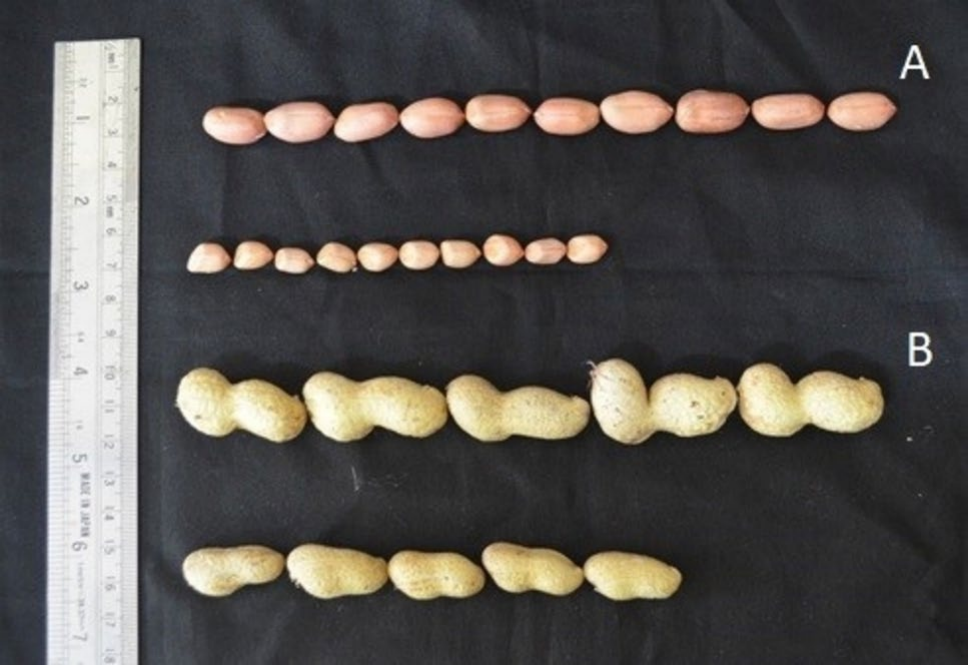
Variation in seed and pod size of mutant, TG 89 (up) and its parent, TG 26 (down). *A*  Seed size variation, *B*  Pod size variation

**Fig. 2:**
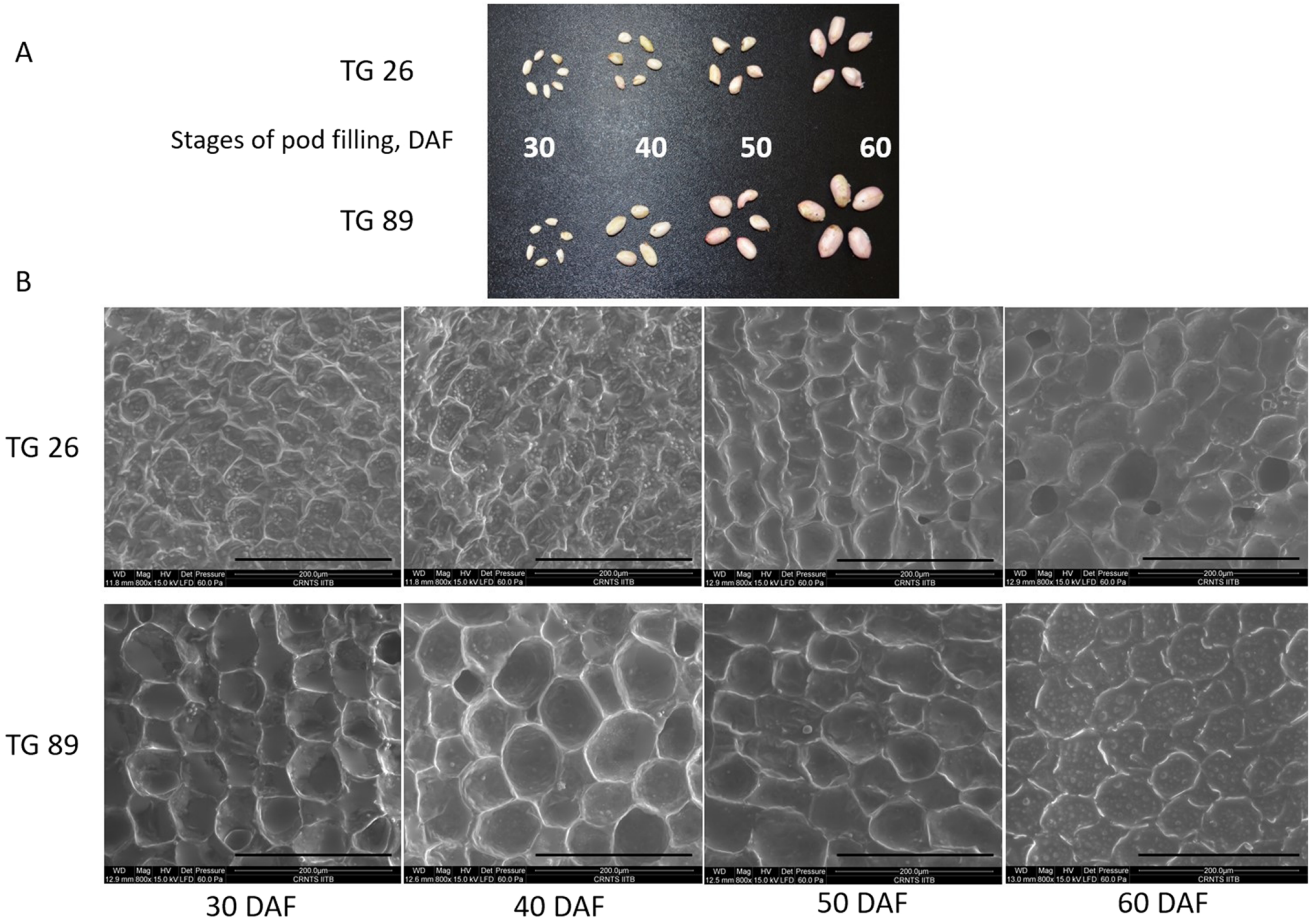
**A** Variation in seed size of parent, TG 26 and mutant, TG 89 at four different stages of pod filling. **B** ESEM images showing differences in cell area of transverse seed cotyledon sections of parent, TG 26 and mutant, TG 89 at four developmental stages. *DAF*  Days after flowering, Scale bars: 200 μm

**Fig. 3:**
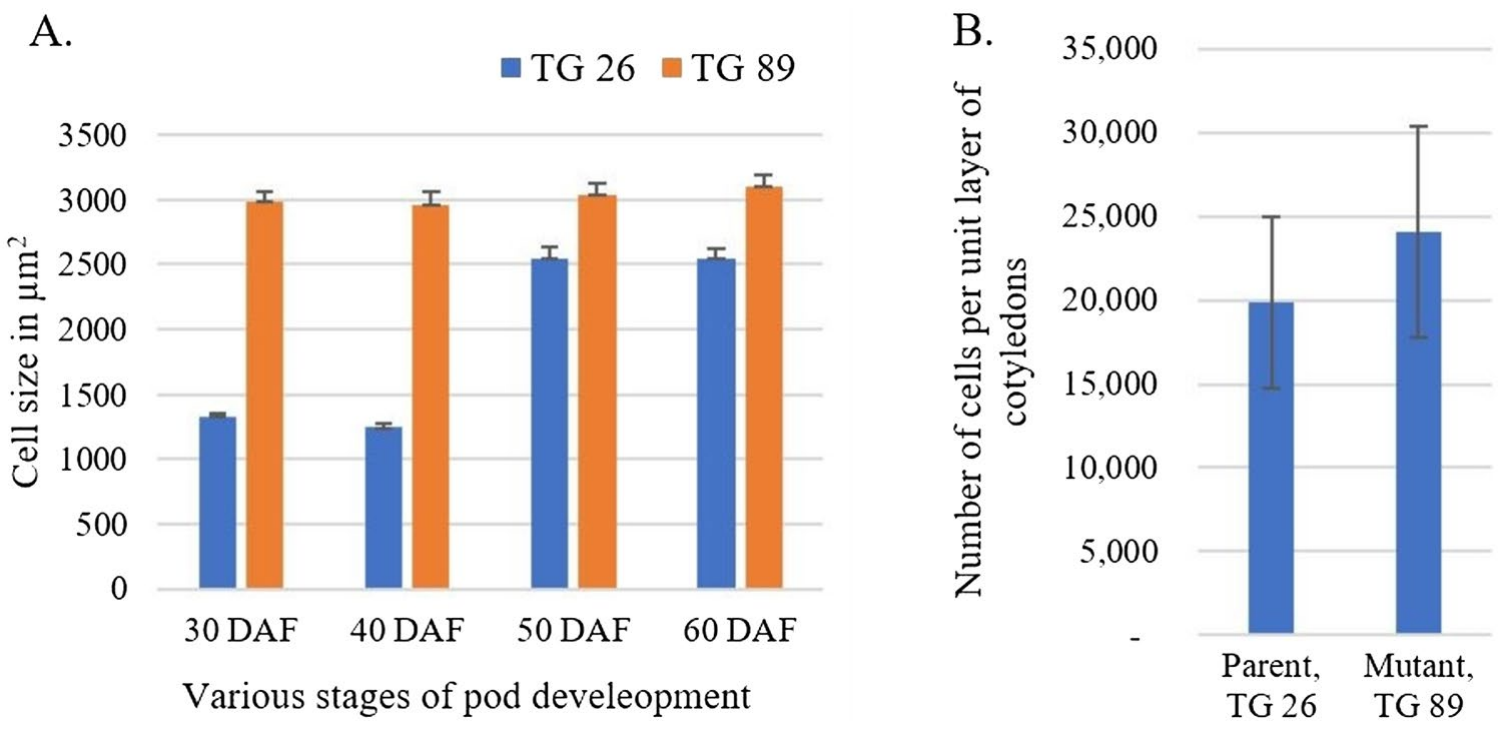
Variation in cell size (µm^2^) and cell number per unit layer of cotyledon in mutant TG 89 and its parent TG 26. **A**  Variation for cell size; **B**   Variation in number of cells, *DAF*  Days after flowering

Field experiments revealed that TG 26 and TG 89 have HKW of 40.0–48.0 g and HKW of 75.0–82.0 g, respectively. Thus, TG 89 has 76.7% more HKW than its parent and has significantly larger pod size (Fig. 1). To understand the histological development of the large seed, the developing pods of the mutant and its parent were harvested at 30, 40, 50 and 60 DAF and were used for environmental scanning electron microscopy (ESEM). ESEM images revealed that the majority of the tissue of the cotyledon was made up of nearly isodiametric, parenchyma cells (Fig. 2). The cell area of the mutant at various development stages was significantly larger than the parent. Growth of organs in plants occurs through an initial proliferative phase where the number of cells increases, followed by an increase in cell size to its maximum set size. The larger cell area in the large seed mutant, TG 89 was observed right from 30 DAF, whereas the cell area in the parent has reached its maximum size at 50 DAF (Fig. 3A). The mean cell area of cotyledon cells of the mutant was 3023 µm^2^ and parent was 1916 µm^2^. Although the area of the unit layer of cotyledons was significantly larger in mutant (0.827 cm^2^) than its parent (0.398 cm^2^), the mean difference between the number of cells per unit layer of cotyledon was insignificant [*t* (df 120) = 1.896, *p* > 0.05], indicating that the number of cells in mutant and parent seeds remained the same (Fig. 3B). Similar results on increase in cell size of cotyledons in induced large seed mutants in groundnut has been reported earlier (Joshua and Bhatia 1983; Sung and Chen 1990). Sung and Chen (1990) reported that cell size in cotyledons begins to increase only when the number of cells in cotyledon approaches their maxima. Malladi and Hirst (2010) reported enhanced fruit size in an apple Gala mutant was facilitated by increased cell size. Cheniclet et al. (2005) have also reported that cell size was positively associated with fruit size in tomato, therefore, increased cell size may be one of the significant factors for large pods in the mutant TG 89. However, Bohner and Bangerth (1988) reported an increase in cell number in an induced large fruit mutant of tomato governed by high concentrations of cytokinins which were correlated with high cell division activity. Zhang et al. (2013) demonstrated the regulatory role of an MYB56 transcription factor in increasing seed size of a large seed mutant of *Arabidopsis thaliana* by increasing the number of cells in its integument.

### BSA identified putative linkage of three markers with large seed trait in TG 89

Identification of molecular markers is important to transfer the economic trait into high-yielding genotypes in an efficient manner. BSA was used for the identification of markers linked to target traits in specific regions of the genome (Michelmore et al. 1991). Of the 680 Miniature Inverted-repeat Transposable Element (MITE) and 751 Simple Sequence Repeat (SSR) markers, 64 (14.58%) MITE and 19 (4.15%) SSR markers, respectively were found polymorphic between TG 89 and ICGV 15007. A higher amount of polymorphism for MITE markers was also earlier reported in groundnut (Hake et al. 2017b). The low level of polymorphism for SSR markers was due to the usage of large proportions of EST-SSR markers in this study (Mondal et al. 2012). These polymorphic 64 MITE and 19 SSR markers were employed for bulk segregant analysis and three markers (AhTE333, AhTE278 and Ah1TC3A12) were found to be completely discriminating the two bulks (Suppl. Figure 1).

### QTL analysis detected an additive QTL for mutant large seed trait in the A05 chromosome

A total of 85 polymorphic markers comprising of 19 SSR, 64 MITE markers and two allele-specific markers for fatty acid desaturase gene were utilized for the genotyping of the whole F_2_ mapping population towards the construction of a genetic linkage map. Out of the 85 polymorphic markers, 52 were codominant and 33 were dominant in their amplification reactions. Out of 52 codominant markers, 38 showed segregation ratios of 1:2:1 remaining 14 markers showed segregation distortion. Of the 33 dominant markers, 28 showed segregation ratios of 3:1. Linkage mapping generated 14 linkage groups with a total genetic map distance of 1053 cM wherein, 12 markers remained unlinked. The lowest number of markers (three) were found on linkage groups 1, 3, 9 and 10. The maximum number (16) of markers were populated on linkage group 5. Of the 16 markers mapped in linkage group 5, 10 were distorted in nature. The shortest linkage group was linkage group 1 with two markers which were tightly linked. The average map distance between the two markers ranged from 3.1 cM on linkage group 13 to 31.6 cM on linkage group 5. Chromosomal references for the linkage group were determined by comparison of the location of markers with the known groundnut consensus map (Shirasawa et al. 2012; Hake et al. 2017a; Lu et al. 2018). Accordingly, linkage group 4 was named as the A05 chromosome. Of the eight markers mapped in the A05 chromosome, only A05S812 was distorted in nature.

**Fig. 4:**
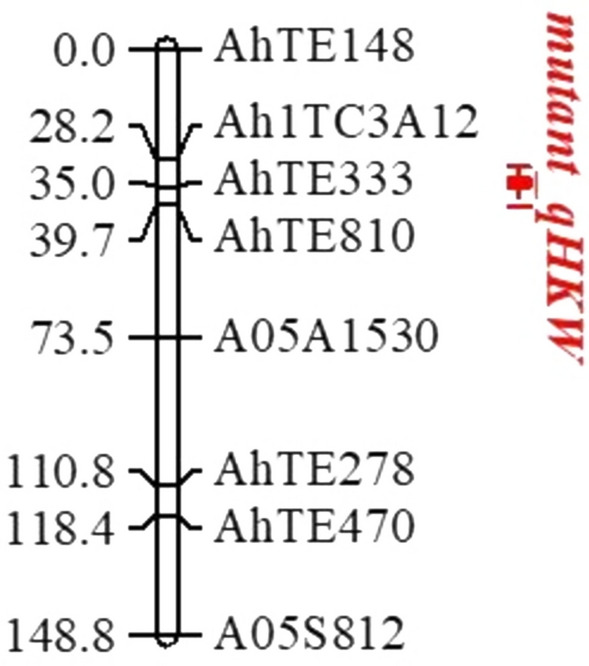
Linkage map depicting position of QTL for mutant large seed trait in A05 chromosome of cultivated groundnut

Phenotyping of the parents and mapping population showed that ICGV 15007 and TG 89 have HKW of 35.5–40.0 g and HKW of 75.0–82.0 g, respectively. To map the mutant large seed trait, mutant, TG 89 (large seed) was crossed with ICGV 15007 (a distant normal seed genotype) to develop an F_2_ mapping population consisting of 122 plants segregating for the large seed trait. The *F*_2_ mapping population showed normal distribution for seed size, ranging from 30 to 75 g with a mean of 51.9 g (Shapiro–Wilk test, *W* = 0.98, *P* = 0.03). In QTL analysis, LOD thresholds for phenotyping data were found to be 3.36. ICIM analysis revealed one significant QTL (LOD value = 4.08) for HKW (mutant_qHKW) with a PVE of 12.7% located on the A05 linkage group (Fig. 4). The mutant_qHKW was located between two flanking markers AhTE333 and AhTE810 spanning a map distance of 4.7 cM. The mutant_qHKW had its peak at 35.5 cM with a confidence interval 32.5 to 39.5 cM and it has an additive value of 4.9 g. While the dominance effect of the QTL is only 0.39 g. Though marker Ah1TC3A12 was also BSA positive, it was located fairly outside the QTL confidence interval. Thus, the marker AhTE333 was tightly linked with the large seed trait. This region could be targeted for further fine mapping of genes responsible for the large seed trait. The localization of the major QTL for this mutant large seed trait was consistent with the finding of Luo et al. (2018) that the pod weight and seed size QTLs were located on the A05 chromosome. The main additive QTL ‘mutant_qHKW’ was present in a map interval surrounded by MITE markers AhTE333 and AhTE810. This map interval corresponded to 90.65 to 107.24 Mbp in the A05 chromosome (based on BLASTn of source GenBank sequence of AhTE333 and AhTE810 in peanutbase). This region harbours a putative gene for mutant phenotypes and codes for protein *Arahy.5M7JWE* having TIFY 6B domain. This TIFY domain is a conserved amino acid sequence for a variety of plant transcription factors and is also conserved in *BIG SEEDS 1* (*BS1*). BLASTp search of this BS 1 protein from *Arabidopsis lyrata* (AKN91666) detected ten orthologs in the *Arachis hypogaea* genome (https://peanutbase.org/blast) (Table 1). One such ortholog *Arahy.5M7JWE* was present in the A05 chromosome at the position of 102,476,137 to 102,481,084 bp that was perfectly placed in between the map interval (90.65 to 107.24 Mbp) of the identified ‘mutant_qHKW’ in A05 chromosome.

**Table 1:** Distribution of BIG SEEDS 1 protein orthologs over cultivated groundnut genome

	Orthologs protein	Chromosome	Identity	Positive coverage	*P* value
1	Arahy.EXPS8P (Protein TIFY-4B like isoform X1)	Arahy.19	41.72%	56.21%	2.22 × e^−62^
2	Arahy.EFG4M9 (jasmonate-zim-domain protein 1)	Arahy.13	35.88%	48.85%	9.15 × e^−5^
3	Arahy.BEZ7GQ (jasmonate-zim-domain protein 1)	Arahy.03	36.94%	49.55%	1.03 × e^−7^
4	Arahy.CDP1KS (Protein TIFY-4B like isoform X)	Arahy.13	27.61%	42.94%	2.15 × e^−7^
5	**Arahy.5M7JWE (Protein TIFY-6B like isoform X3)**	**Arahy.05**	**27.52%**	**46.31%**	**4.59 × e**^**−7**^
6	Arahy.23LT4W (Protein TIFY-68 like isoform)	Arahy.15	27.52%	46.31%	6.03 × e^−7^
7	Arahy.76C240 (jasmonate-zim-domain protein 1)	Arahy.01	34.34%	47.47%	1.26 × e^−6^
8	Arahy.I4L3EJ (jasmonate-zim-domain protein 6)	Arahy.09	32.11%	52.29%	1.79 × e^−6^
9	Arahy.WAJ05F (protein TIFY 6B-like isoform X3)	Arahy.03	26.38%	41.72%	6.66 × e^−6^
10	Arahy.NLIW19 (jasmonate-zim-domain protein 6)	Arahy.19	33.94%	51.38%	6.85 × e^−6^

The row in bold font indicates the identified orthologs of BIG SEEDS 1 inside the identfied QTL interval in A05 chromosome of groundnut

### A *BS1* ortholog of groundnut in A05 chromosome is the putative candidate gene for mutant large seed trait

Loss of function mutation in the *BS1* gene has been shown to control seed size and pod size in *Medicago trunculata* and soybean (Ge et al. 2016). *BS1* encodes a plant-specific transcription factor that belongs to a family of transcriptional regulators. These transcriptional regulators plays a key role in the control of the size of plant lateral organs, including seeds, seed pods and leaves, through a regulatory module called PEAPOD pathway that targets primary cell proliferation. Down-regulation of soybean *BS1* gene using an artificial microRNA also resulted in increased size of seeds, seed pods and leaves, thus revealing a key and conserved role of BS1 in the control of organ size in legumes (Ge et al. 2016). Certain growth-regulatory networks which plays a key role in determining organ size, are highly conserved in eudicot species, represented by the PEAPOD (PPD)/KINASE-INDUCIBLE DOMAIN INTERACTING (KIX) /STERILE APETALA (SAP) module regulating critical jasmonate pathway (Schneider et al. 2021). This PEAPOD (PPD) pathway involves transcriptional regulators like PPD1 and PPD2 that, together with the JASMONATE ZIM-DOMAIN (JAZ) proteins and TIFY DOMAIN PROTEIN 8 (TIFY8), constitute the plant-specific class II TIFY protein family. These proteins control the primary cell cycle arrest which is reflected in the arrest of pavement cell divisions (Baekelandt et al. 2018). BS1 belongs to these TIFY family of proteins, that act as transcription corepressors. These corepressors interact with Novel Interactor of JAZ (NINJA), TOPLESS (TPL) and TOP-LESS-RELATED PROTEINs (TPRs) to suppress downstream gene expression in jasmonate signalling pathway (Cuellar et al. 2014). In plants, a transcriptional regulator STERILE APETALA-like protein (SAP protein) is also known to control plant organ size including seeds. This SAP protein is an F-box family protein which is one of the components of a SKP1/Cullin/F-box (SCF complex) E3 ubiquitin ligase complex, which controls flower, fruit and leaf size in *Arabidopsis*. This SAP protein increases cell proliferation by physically associating with the repressor factors PPDs (PEAPOD1 and PEAPOD2) or other PEAPOD orthologs (like BS1 in legume) and targeting them for degradation (Wang et al. 2016; Li et al. 2018). Loss of function mutation in the *SAP* gene causes severe anomalies in inflorescence, flower and ovule development, leading to yield penalty (Byzova et al. 1999). Since the mutant TG 89 has a bigger seed size along with the increment of leaf size, the probable loss of function mutation in *BS 1* gene in TG 89 may determine the increased seed size as evident in soybean and *Medicago trunculata* (Ge et al. 2016). The levels of mRNA expression of *arahy.5M7JWE* genes were estimated by real-time PCR in mutant (TG 89) vs. parent (TG 26) which indicated that *arahy.5M7JWE* was significantly downregulated (0.44-fold) in the mutant, this indicates that the recessive mutation in this gene might be responsible for phenotype change in mutant. This gene could be a probable target for reverse genetics approach like Targeting Induced Local Lesions IN Genomes (TILLING) for induction of large seed mutant.

### Flanking markers of ‘mutant_qHKW’ show promise in marker-assisted selection

**Fig. 5:**
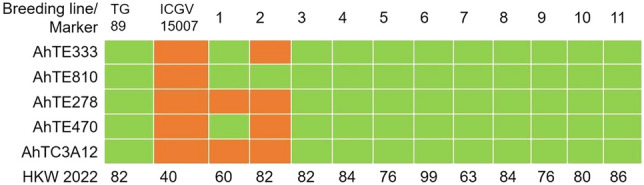
Validation of high-yielding breeding lines with mutant_qHKW flanking markers from linkage group A05. Green colour depicts for mutant type and orange colour depicts for parent type alleles

Further, to validate the ‘mutant_qHKW’ QTL, screening of flanking markers AhTE333 and AhTE810 on a set of 11 high-yielding breeding lines (*F*_7_ generation) which were developed by utilizing TG 89 as one of the parents (as a source of large seed trait), revealed 10 lines (90.9%) had the correct classification of linked marker reaction for mutant derived large seed trait (Fig. 5). When analysed these 11 lines with 5 closely linked markers in A05, 9 breeding lines conserved entire genomic segment from mutant type and has high HKW value while 1 had retained only mutant_qHKW flanking markers (Fig. 5). This tagging of the HKW locus with MITE markers will be useful in the rapid selection of genotypes with the better HKW from segregating populations involving large seed mutant in hybridization. Such validation of QTL with linked markers is crucial in groundnut breeding programs because it provides precise and efficient genetic markers to expedite the selection process for the seed size trait. These markers can help in mitigating pleiotropic effects by allowing selection of target-specific loci associated with desirable traits by minimizing the unintended influence on unrelated traits.

## Conclusion

In conclusion, the present work revealed that the large seed size in TG 89 evolved due to the increment of the size of seed cotyledonary cells. Further, QTL analysis detected a major QTL of mutant origin that explains 12.7% of phenotypic variation due to the seed size variation in the F_2_ population. The identified QTL corresponds to 90.65 to 107.24 Mbp on the A05 chromosome, where an ortholog of *BIG SEEDS 1* (*BS 1*) exists in the cultivated groundnut genome. The *BS 1 is a* negative regulator that controls seed size and pod size. In future, fine mapping and map-based isolation of orthologs of *BS 1* gene can be done to exploit such valuable mutation through new breeding technologies. Present study also reports identification and validation of molecular markers flanking the QTL region that will be helpful in expediting trait identification by offering early, precise and efficient selection in groundnut breeding. This precision in selection will contribute to the development of improved groundnut genotypes with larger seed size, directly contributing to increased crop yield.

## Supplementary Information

Below is the link to the electronic supplementary material.

Supplementary file1 (DOCX 131 KB)

## Data Availability

The manuscript contains all the relevant data within it either in main text or supplement informations.
