# Correction: Molecular tagging of seed size using MITE markers in an induced large seed mutant with higher cotyledon cell size in groundnut

**DOI:** 10.1007/s13205-026-04970-1

**Published:** 2026-07-08

**Authors:** Poonam Gajanan Bhad, Suvendu Mondal, Anand M. Badigannavar

**Affiliations:** 1 Nuclear Agriculture and Biotechnology Division, Bhabha Atomic Research Centre, Mumbai, 400085 India; 2 Homi Bhabha National Institute, Training School Complex, Anushaktinagar, Mumbai, 400094 India

**Correction: 3 Biotech 14:56 (2024)** 10.1007/s13205-023-03909-0

The article “Molecular tagging of seed size using MITE markers in an induced large seed mutant with higher cotyledon cell size in groundnut” written by Poonam Gajanan Bhad, Suvendu Mondal, Anand M. Badigannavar was originally published electronically on the publisher’s internet portal on 29th January 2024 without open access. With the author(s)’ decision to opt for Open Choice the copyright of the article changed on 10th July 2026 to © The Author(s) 2026 and the article is forthwith distributed under a Creative Commons Attribution 4.0 International License, which permits use, sharing, adaptation, [50] distribution and reproduction in any medium or format, as long as you give appropriate credit to the original author(s) and the source, provide a link to the Creative Commons licence, and indicate if changes were made. The images or other third party material in this article are included in the article’s Creative Commons licence, unless indicated otherwise in a credit line to the material. If material is not included in the article’s Creative Commons licence and your intended use is not permitted by statutory regulation or exceeds the permitted use, you will need to obtain permission directly from the copyright holder. To view a copy of this licence, visit http://creativecommons.org/licenses/by/4.0.

The original article has been corrected.

