# Supplementary material for: Molecular tagging of seed size using MITE markers in an induced large seed mutant with higher cotyledon cell size in groundnut
Source: 3 Biotech. 2024 Jan 29;14(2):56. doi: 10.1007/s13205-023-03909-0 (PMC10825088; doi:10.1007/s13205-023-03909-0)
Supplement: Supplementary file 1 — Supplementary file1 (DOCX 131 KB) [file 13205_2023_3909_MOESM1_ESM.docx]

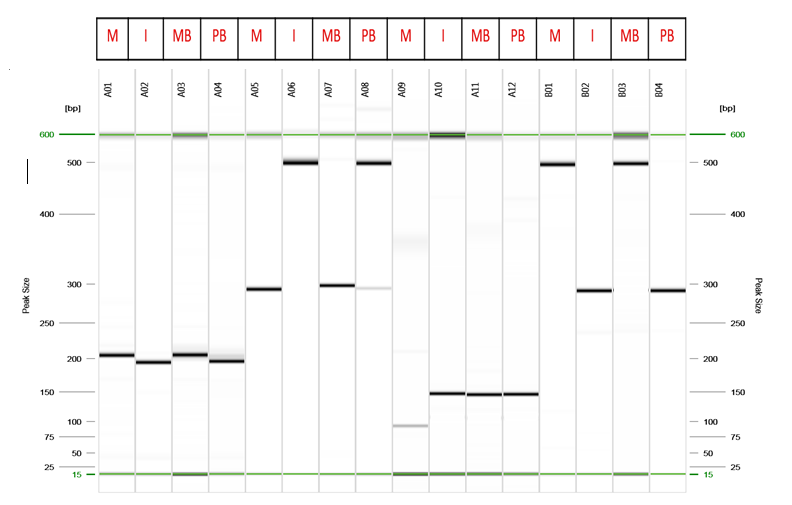


**Suppl. Fig 1:** Capillary gel electrophoresis representation of bulk segregant analysis of Mutant (M), distant parent (I), mutant bulk (MB) and distant parent bulks (PB) from the F_2_ cross TG 89 X ICGV 15007.
Note: Marker order 1-4. Ah1TC3A12, 5-8. AhTE333, 9-12. AhTE810, 12-16 AhTE278.
